# Supplementary material for: The effect of modulating the quantity of enzymes in a model ethanol pathway on metabolic flux in Synechocystis sp. PCC 6803
Source: PeerJ. 2019 Aug 28;7:e7529. doi: 10.7717/peerj.7529 (PMC6717505; doi:10.7717/peerj.7529)
Supplement: Supplemental Information 1 [file peerj-07-7529-s001.docx]

**Supplement**

**Supplementary Table 1.** BASIC- compatible primers used in this study (BASIC prefix and suffix sequences in bold)

| **DNA part** | **Forward primer (prefix)** | **Reverse primer (suffix)** | **source** |
| --- | --- | --- | --- |
| 5’-NS (neutral site slr0168) | **TCTGGTGGGTCTCTGTCC**CCTTTGACAACAATGTGGCCTGG | **CGATAGGTCTCCCGAGCC**CCATATAAATCCCCGCCACTGTTATTTTG | *Synechocystis* 6803 genomic DNA |
| 3’-NS (neutral site slr0168) | **TCTGGTGGGTCTCTGTCC**AGACCAAGCCCAATTTCGTTTGC | **CGATAGGTCTCCCGAGCC**GCTAAACCCACCTCTTGCCCAATG | *Synechocystis* 6803 genomic DNA |
| PA1lacO1 | **TCTGGTGGGTCTCTGTCC**GACACCATCGAATGGTGCAAAACC | **CGATAGGTCTCCCGAGCC**AATTGTTATCCGCTCACAATTGAATCTAAGTATC | pDF-lac (Guererro et al, 2012) |
| eYFP | **TCTGGTGGGTCTCTGTCC**ATGGTGAGCAAGGGCGAGGAG | **CGATAGGTCTCCCGAGCC**TTACTTGTACAGCTCGTCCATGCCG | vector provided by Dr. P. Armshaw, UL, Limerick, Ireland |
| SpecR | **TCTGGTGGGTCTCTGTCC**ATTCTCACCAATAAAAAACGCCCG | **CGATAGGTCTCCCGAGCC**ATGACATGTTTTTTTGGGGTACAGTCTATG | pDF-lac (Guerrero et al., 2012) |
| Z. mobilis  pyruvate decarboxylase (pdc) | **TCTGGTGGGTCTCTGTCC**ATGAGTTATACTGTCGGTACCTATTTAGCGG | **CGATAGGTCTCCCGAGCC**CTAGAGGAGCTTGTTAACAGGCTTACGG | pPSBA2EtoHKan provided by Dr. P. Armshaw, UL, Limerick, Ireland |
| alcohol dehydrogenase -Synechocystis sp. PCC 6803 slr1192 (adh) | **TCTGGTGGGTCTCTGTCC**ATGATTAAAGCCTACGCTGCCCTG | **CGATAGGTCTCCCGAGCC**CTAATTTTTACTATGGCTGAGCACTACCCG | pPSBA2EtoHKan provided by Dr. P. Armshaw, UL, Limerick, Ireland |
| pdc mutagenesis primers | GCGGCTTGTCCAGATTGGCCTCAAGCATCACTTCGCAG | CTGCGAAGTGATGCTTGAGGCCAATCTGGACAAGCCGC | IDT |
| *E. coli* pyruvate kinase (pykF) | **TCTGGTGGGTCTCTGTCC**ATGAAAAAGACCAAAATTGTTTGCACCATC | **CGATAGGTCTCCCGAGCC**TTACAGGACGTGAACAGATGCGG | *E. coli* genomic DNA |
| bifunctional fructose-1,6-bisphosphatase/sedoheptulose-1,7-bisphosphatase, *slr2094 (bibp)* | **TCTGGTGGGTCTCTGTCC**GTGGACAGCACCCTCGGTTTAG | **CGATAGGTCTCCCGAGCC**TTAATGCAGTTGGATTACTTTGGGGc | *Synechocystis* 6803 genomic DNA |

**Supplementary Table 2**. Ribosome binding site sequences used in this study. The capitalized nucleotides refer to the spacer sequence and are not part of the RBS.

| **RBS** | **RBS sequence** | **BioBrick™ part number** | **Additional information** |
| --- | --- | --- | --- |
| **RBS A** | AtcacacaggacTA | BBa_B0033 |  |
| **RBS B** | AaagaggggaaaTA | BBa_B0064 |  |
| **RBS C** | AaagaggagaaaTA | BBa_B0034 |  |
| **RBS D** | AtcacaaggaggTA | - | *E. coli* consensus Shine-Dalgarno sequence |
| **RBS E** | AttagtggaggtTA | - | RBS* in Heidorn et al., 2011 |

**Supplementary Table 3. Plasmid DNA constructed in this study.**

| **Strain** | **Plasmid(s)** | **Genotype** |
| --- | --- | --- |
|  | CB1 | pMB1, AmpR, PA1lacO1 promoter, GFP placeholder, slr0168 integration site, SpecR |
|  | NB2 | pMB1, AmpR, Ptrc promoter, GFP placeholder, slr1395 integration site, ChlR |
| RBSA_eYFP | CB1_RBSA_eYFP | CB1, RBS A, eYFP |
| RBSB_eYFP | CB1_RBSB_eYFP | CB1, RBS B, eYFP |
| RBSC_eYFP | CB1_RBSC_eYFP | CB1, RBS C, eYFP |
| RBSD_eYFP | CB1_RBSD_eYFP | CB1, RBS D, eYFP |
| RBSE_eYFP | CB1_RBSE_eYFP | CB1, RBS E, eYFP |
| AA | CB1_RBSA_pdc_RBSA_adh | CB1, RBS A, pdc, RBS A, adh |
| AB | CB1_RBSA_pdc_RBSB_adh | CB1, RBS A, pdc, RBS B, adh |
| AC | CB1_RBSA_pdc_RBSC_adh | CB1, RBS A, pdc, RBS C, adh |
| AD | CB1_RBSA_pdc_RBSD_adh | CB1, RBS A, pdc, RBS D, adh |
| AE | CB1_RBSA_pdc_RBSE_adh | CB1, RBS A, pdc, RBS E, adh |
| BA | CB1_RBSB_pdc_RBSA_adh | CB1, RBS B, pdc, RBS A, adh |
| BB | CB1_RBSB_pdc_RBSB_adh | CB1, RBS B, pdc, RBS B, adh |
| BC | CB1_RBSB_pdc_RBSC_adh | CB1, RBS B, pdc, RBS C, adh |
| BD | CB1_RBSB_pdc_RBSD_adh | CB1, RBS B, pdc, RBS D, adh |
| BE | CB1_RBSB_pdc_RBSE_adh | CB1, RBS B, pdc, RBS E, adh |
| CA | CB1_RBSC_pdc_RBSA_adh | CB1, RBS C, pdc, RBS A, adh |
| CB | CB1_RBSC_pdc_RBSB_adh | CB1, RBS C, pdc, RBS B, adh |
| CC | CB1_RBSC_pdc_RBSC_adh | CB1, RBS C, pdc, RBS C, adh |
| CD | CB1_RBSC_pdc_RBSD_adh | CB1, RBS C, pdc, RBS D, adh |
| CE | CB1_RBSC_pdc_RBSE_adh | CB1, RBS C, pdc, RBS E, adh |
| DB | CB1_RBSD_pdc_RBSB_adh | CB1, RBS D, pdc, RBS B, adh |
| DC | CB1_RBSD_pdc_RBSC_adh | CB1, RBS D, pdc, RBS C, adh |
| DD | CB1_RBSD_pdc_RBSD_adh | CB1, RBS D, pdc, RBS D, adh |
| DE | CB1_RBSD_pdc_RBSE_adh | CB1, RBS D, pdc, RBS E, adh |
| EA | CB1_RBSE_pdc_RBSA_adh | CB1, RBS E, pdc, RBS A, adh |
| EB | CB1_RBSE_pdc_RBSB_adh | CB1, RBS E, pdc, RBS B, adh |
| EC | CB1_RBSE_pdc_RBSC_adh | CB1, RBS E, pdc, RBS C, adh |
| ED | CB1_RBSE_pdc_RBSD_adh | CB1, RBS E, pdc, RBS D, adh |
| EE | CB1_RBSE_pdc_RBSE_adh | CB1, RBS E, pdc, RBS E, adh |
| A | CB1_RBSA_pdc | CB1, RBS A, pdc |
| B | CB1_RBSB_pdc | CB1, RBS B, pdc |
| C | CB1_RBSC_pdc | CB1, RBS C, pdc |
| D | CB1_RBSD_pdc | CB1, RBS D, pdc |
| E | CB1_RBSE_pdc | CB1, RBS E, pdc |
| EE_RBS A PYK | CB1_RBSE_pdc_RBSE_adh_RBSA_pyk | CB1, RBS E, pdc, RBS E, adh, RBSA, pyk |
| EE_RBS D PYK | CB1_RBSE_pdc_RBSE_adh _RBSD_pyk | CB1, RBS E, pdc, RBS E, adh, RBSD, pyk |
| EE_RBS E PYK | CB1_RBSE_pdc_RBSE_adh _RBSE_pyk | CB1, RBS E, pdc, RBS E, adh, RBSE, pyk |
| EE_RBS A BiBP | CB1_RBSE_pdc_RBSE_adh_RBSA_bibp | CB1, RBS E, pdc, RBS E, adh, RBSA, bibp |
| EE_RBS B BiBP | CB1_RBSE_pdc_RBSE_adh_RBSB_bibp | CB1, RBS E, pdc, RBS E, adh, RBSB, bibp |
| EE_RBS C BiBP | CB1_RBSE_pdc_RBSE_adh_RBSC_bibp | CB1, RBS E, pdc, RBS E, adh, RBSC, bibp |
| EE_RBS D BiBP | CB1_RBSE_pdc_RBSE_adh_RBSD_bibp | CB1, RBS E, pdc, RBS E, adh, RBSD, bibp |
| EE_AA | CB1_RBSE_pdc_RBSE_adh  NB2_RBSA_pyk_RBSA_bibp | CB1, RBS E, pdc, RBS E, adh, NB2, RBS A, pyk, RBS A, bibp |
| EE_AB | CB1_RBSE_pdc_RBSE_adh  NB2_RBSA_pyk_RBSB_bibp | CB1, RBS E, pdc, RBS E, adh, NB2, RBS A, pyk, RBS B, bibp |
| EE_AD | CB1_RBSE_pdc_RBSE_adh  NB2_RBSA_pyk_RBSD_bibp | CB1, RBS E, pdc, RBS E, adh, NB2, RBS A, pyk, RBS D, bibp |
| EE_BB | CB1_RBSE_pdc_RBSE_adh  NB2_RBSB_pyk_RBSB_bibp | CB1, RBS E, pdc, RBS E, adh, NB2, RBS B, pyk, RBS B, bibp |
| EE_DA | CB1_RBSE_pdc_RBSE_adh  NB2_RBSD_pyk_RBSA_bibp | CB1, RBS E, pdc, RBS E, adh, NB2, RBS D, pyk, RBS A, bibp |
| EE_DD | CB1_RBSE_pdc_RBSE_adh  NB2_RBSD_pyk_RBSD_bibp | CB1, RBS E, pdc, RBS E, adh, NB2, RBS D, pyk, RBS D, bibp |
| EE_DE | CB1_RBSE_pdc_RBSE_adh  NB2_RBSD_pyk_RBSE_bibp | CB1, RBS E, pdc, RBS E, adh, NB2, RBS D, pyk, RBS E, bibp |
| EE_EA | CB1_RBSE_pdc_RBSE_adh  NB2_RBSE_pyk_RBSA_bibp | CB1, RBS E, pdc, RBS E, adh, NB2, RBS E, pyk, RBS A, bibp |

**Supplementary Table 4. Signature peptides and transtions for SRM Mass spectroscopy.**

| **Protein** | **Peptide** | **Mass info** | **Retention time** |
| --- | --- | --- | --- |
| pdc | LVQIGLK.+2y6.light | 385.8 / 657.4 | 12,2 |
| pdc | LVQIGLK.+2y5.light | 385.8 / 558.4 | 12,2 |
| pdc | LVQIGLK.+2y4.light | 385.8 / 430.3 | 12,2 |
| pdc | VAVLVGSK.+2y6.light | 386.8 / 602.4 | 8,8 |
| pdc | VAVLVGSK.+2y5.light | 386.8 / 503.3 | 8,8 |
| pdc | VAVLVGSK.+2y4.light | 386.8 / 390.2 | 8,8 |
| pdc | TGALDFFK.+2y6.light | 449.7 / 740.4 | 17 |
| pdc | TGALDFFK.+2y5.light | 449.7 / 669.4 | 17 |
| pdc | TGALDFFK.+2y4.light | 449.7 / 556.3 | 17 |
| pdc | TGGELAEAIK.+2y8.light | 494.8 / 830.5 | 9,7 |
| pdc | TGGELAEAIK.+2y6.light | 494.8 / 644.4 | 9,7 |
| pdc | TGGELAEAIK.+2y5.light | 494.8 / 531.3 | 9,7 |
| adh | AWGC[CAM]EVTAFTSSAR.+2y10.light | 771.9 / 1068.5 | 15,1 |
| adh | AWGC[CAM]EVTAFTSSAR.+2y9.light | 771.9 / 939.5 | 15,1 |
| adh | AWGC[CAM]EVTAFTSSAR.+2y8.light | 771.9 / 840.4 | 15,1 |
| adh | FDYIISTVNLK.+2y6.light | 656.9 / 661.4 | 18,5 |
| adh | FDYIISTVNLK.+2y10.light | 656.9 / 1165.6 | 18,5 |
| adh | FDYIISTVNLK.+2y9.light | 656.9 / 1050.6 | 18,5 |
| adh | FDYIISTVNLK.+2y8.light | 656.9 / 887.6 | 18,5 |
| adh | FDYIISTVNLK.+2y7.light | 656.9 / 774.5 | 18,5 |
| atpB | VIDLLTPYR.+2y7.light | 545.3 / 877.5 | 17,7 |
| atpB | VIDLLTPYR.+2y6.light | 545.3 / 762.5 | 17,7 |
| atpB | VIDLLTPYR.+2y5.light | 545.3 / 649.4 | 17,7 |
| atpB | YVSLADTIK.+2y8.light | 505.3 / 846.5 | 13,3 |
| atpB | YVSLADTIK.+2y7.light | 505.3 / 747.4 | 13,3 |
| atpB | YVSLADTIK.+2y6.light | 505.3 / 660.4 | 13,3 |
| atpB | YVSLADTIK.+2y5.light | 505.3 / 547.3 | 13,3 |
| **collision** |  |  |  |
